# No compelling evidence for early small-scale animal husbandry in Atlantic NW Europe

**DOI:** 10.1038/s41598-022-05073-6

**Published:** 2022-01-26

**Authors:** Nathalie Ø. Brusgaard, Canan Çakirlar, Michael Dee, Merita Dreshaj, Jolijn Erven, Hans Peeters, Daan Raemaekers

**Affiliations:** 1 Groningen Institute of Archaeology, University of Groningen, Poststraat 6, 9712 ER Groningen, The Netherlands; 2 Center for Isotope Research, University of Groningen, Nijenborgh 6, 9747 AG Groningen, The Netherlands

**Keywords:** Anthropology, Archaeology

**ARISING FROM**: P. Crombé et al.; *Scientific Reports* 10.1038/s41598-020-77002-4 (2020).

Crombé et al.^1^ present data which in their opinion provide indications for local animal husbandry in the northwest European lowlands as early as 4800/4600 cal BC. Their argument is built on radiocarbon (^14^C) and stable isotope analyses of bones attributed to sheep/goat and cattle from the site of Bazel “Sluis”, northwest Belgium. Focussing on the bone remains, we argue that their conclusions are unsupported due to lack of direct evidence for a local origin of the animals and uncertainty about the domestic status of the cattle. We propose that the Bazel assemblage provides important new data for the study of the role of domesticates in the 5th millennium cal BC, but that it does not provide new insights into the timing of incipient animal husbandry outside the loess belt. Instead, it leaves room for multiple models of cultural behaviour.

The assemblage studied by Crombé et al.^1^ consists of new material from Bazel, supplemented by finds published in 2016^2^. They list 23—out of a total of 1415 (identified to species)—bone fragments assigned to sheep and/or goat (*Ovis ammon f. aries/Capra aegagrus f. hircus*) (Crombé et al. Table SI 1). The new material comprises 1 specimen attributed to aurochs (*Bos primigenius*) and 117 to cattle (*Bos taurus*). In contrast, the 2016 assemblage comprises 43 aurochs, 75 cattle, and 39 aurochs/cattle^2^. This discrepancy between the two datasets may be caused by differential preservation, collection methods, or differences in contexts, but without clarifying evidence, it is not possible to exclude inter-analyst bias as a primary factor. Indeed, the morphometric distinction between aurochs and cattle is not straightforward^3^. Crombé et al. recognise the issue of distinguishing between the two species, and yet maintain their identifications whilst excluding *Sus* (i.e. wild and domestic pigs) from their study for precisely this reason.

Using the Logarithmic Size Index (LSI) method, we compared the osteometrics of the entire *Bos* meta-population from Bazel with published data from contemporary hunter-gatherer and farming contexts. The results show that most Bazel individuals are large, comparable with specimens of Ertebølle sites, which consist primarily of aurochs (Fig. 1; Supplementary Data S1). Small specimens constitute only the tail of the Bazel population and need not have been domesticated individuals. Sexual dimorphism must be taken into account when interpreting population size variation, as Crombé et al. also point out. Indeed, palaeogenomic analysis recently demonstrated that small *Bos* metacarpi from the Ertebølle site of Rosenhof, Germany (c. 4900–4700 BC)^4^ and a late 4th millennium cal BC site at Twann, Switzerland^5^ were females with aurochs mtDNA.

**Figure 1:**
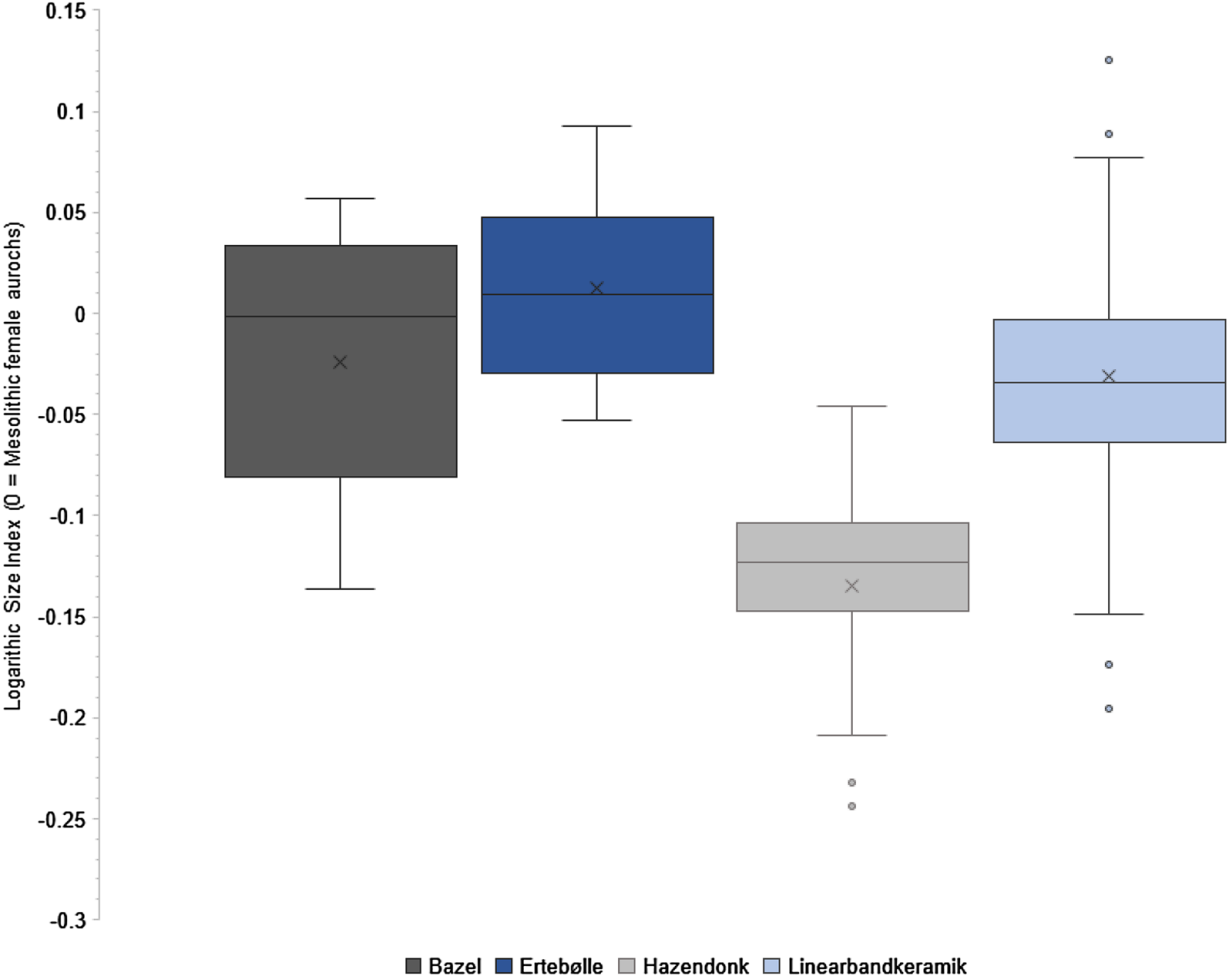
Boxplot showing the Logarithmic Size Indices of *Bos* breadth dimensions from the site Bazel Sluis^1,2^, available Ertebølle culture sites^13^, the Hazendonk culture site of Schipluiden, the Netherlands^13^, and available Linearbandkeramik culture sites^13^ (x = mean).

Alternatively, these smaller specimens could be of domestic origin, brought to the site as body parts, rather than animals on the hoof. This is especially likely for the small metapodial bones presented by Crombé et al. Fleshless metapodial bones (possibly even attached to hides) were frequently used as blanks for tools^6^. Similarly, the sheep/goat could have been transported to the site as butchered parts. Crombé et al. argue that the specimens were likely not imported but instead provide evidence for local animal husbandry based on the δ^13^C and δ^15^N ratios of the *Bos* and sheep/goat bone collagen. This is largely based on their offset from equivalent data from French and German loess sites. In our opinion, this explanation arises from an overinterpretation of the stable carbon and nitrogen isotope results.

Firstly, most of the differences are statistically insignificant, as they fall within the error margins of isotope ratios^7^. Secondly, while the values of some individuals do differ significantly, the individual that deviates the most in its δ^13^C ratios from the French and German sites, also deviates the most from the local red deer δ^13^C ratios from Bazel, demonstrating the complexity of the material. One would need a carbon isoscape of the region to deduce anything meaningful from inter-site differences, the creation of which is a challenge in isotopic research^8^. Lastly, and most importantly, although herbivorous δ^13^C and δ^15^N signatures do reflect diet and therefore, the local environment as Crombé et al. state, it is not a one-to-one relationship. Many individual factors, including age and size, determine the isotopic ratios of an animal^9^.

Consequently, δ^13^C and δ^15^N analysis of bone collagen alone cannot be used to infer an animal's origin. Crombé et al. attempt to substantiate a local origin for the domesticates using Sr isotope analysis. However, without a ^87^Sr/^86^Sr baseline for either the region or site in question, it is not possible to assess whether the individuals are non-local. In fact, four of the individuals exhibit ^87^Sr/^86^Sr signatures that are equally consistent with the Dutch loess and Pleistocene sand areas^10^, which are geographically closer to Bazel than the comparable French and German regions. Moreover, all the Sr analyses were obtained on bones younger than 4300 cal BC, for which local stockbreeding is no longer a matter of discussion^6^. Likewise, the shift in δ^13^C ratios interpreted by the authors as possible evidence for foddering also only applies to the animals from this later period.

The chronological backbone for the argument by Crombé et al. for early animal husbandry is the older cluster of probability distribution of radiocarbon dates, which falls in the first half of the 5th millennium BC (Crombé et al. Table 2, Fig. 3B). It comprises four *Bos*, one goat, and one sheep/goat specimen. While ‘a domestic status is assumed’ by Crombé et al. for the four ‘cattle’ bones (p. 4), this cannot be substantiated by the available evidence, as argued above. The only certain domesticates in this dataset are the two sheep/goat specimens. Their calibrated radiocarbon date ranges are similar to an existent date from Hardinxveld-Giessendam De Bruin (Fig. 2; Table 1); however, the new results are marginally older at 95% probability.

**Figure 2:**
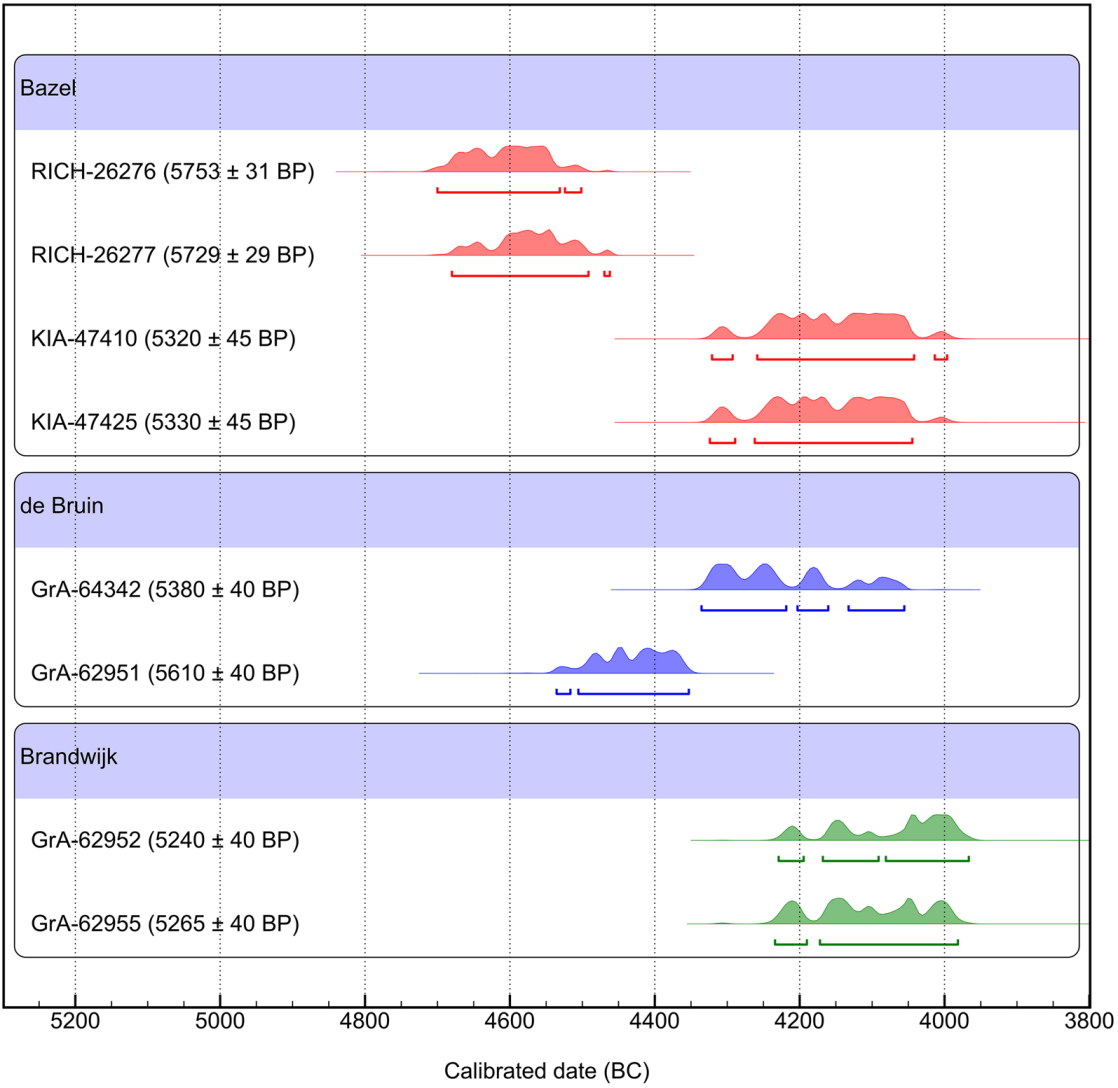
Probability distributions of radiocarbon dates of sheep/goat bones from Bazel Sluis, Hardinxveld-Giessendam de Bruin, and Brandwijk (OxCal v4.4.2^15^). Dates and references are presented in Table 1.

**Table 1:** Radiocarbon dates of sheep/goat bones from Bazel “Sluis”, Hardinxveld-Giessendam de Bruin, and Brandwijk, shown in Fig. 1.

Site	Sample no	Species	Element	δ^13^C (‰ VPDB)	δ^15^N (‰ air)	^14^C results		Publication
						date (year BP)	± (1σ)	
Bazel	KIA-47410	*Ovis ammon f. aries*	cranium	− 23.5	5.3	5320	45	Ervynck et al. 2016^2^
Bazel	KIA-47425	*Ovis ammon f. aries*	calcaneum	− 24.2	5.2	5330	45	Ervynck et al. 2016^2^
Bazel	RICH-26277	*Capra aegagrus f. hircus*	horncore	− 24.0	5.0	5729	29	Crombé et al. 2020^1^
Bazel	RICH-26276	*Ovis ammon f. aries*/*Capra aegagrus f. hircus*	tibia	− 23.1	6.0	5753	31	Crombé et al. 2020^1^
Hardinxveld-Giessendam de Bruin	GRA-64342	*Ovis ammon f. aries*	Metatarsus, distal fused	− 22.5	6.60	5380	40	Çakirlar et al. 2020^6^
Hardinxveld-Giessendam de Bruin	GRA-62951	*Ovis ammon f. aries*/*Capra aegagrus f. hircus*	Radius, proximal half	− 22.9	4.80	5610	40	Çakirlar et al. 2020^6^
Brandwijk	GRA 62952	*Ovis ammon f. aries*/*Capra aegagrus f. hircus*	Upper 3rd molar	− 21.8	9.5	5240	40	Çakirlar et al. 2020^6^
Brandwijk	GRA 62955	*Ovis ammon f. aries*/*Capra aegagrus f. hircus*	Femur, proximal fused	− 22.4	6.6	5265	40	Çakirlar et al. 2020^6^

In conclusion, the significance of the assemblage presented by Crombé et al. rests on the ^14^C dates on the sheep/goat specimens, as two are possibly the oldest examples in the region. The small *Bos* specimens could be domestic cattle, but they are exceptions in the *Bos* assemblage as a whole. There is currently no evidence to support a local origin for the sheep/goat or possible cattle. Although Crombé et al. introduce the assemblage as presenting ‘possible’ animal husbandry, they conclude by saying that ‘the isotope data, although not yet fully conclusive, seems to be in favor of small-scale husbandry from the very beginning’ (p. 11). We argue that the stable isotope results are inconclusive on this point and do not support this assertion.

In our opinion, the existent picture of the northwest European lowlands in the early 5th millennium cal BC therefore remains largely unchanged, and at least two other forms of cultural interaction can explain the evidence presented by Crombé et al.: (gift) exchange between (ceramic) hunter-gatherers and farmers, and transhumance by farmers into hunter-gatherer land beyond the agro-pastoral ‘frontier’. The Bazel data, including the presence of non-local ceramic and lithic artefacts^11^, seem to support a gift-exchange scenario. As such, the data presented by Crombé et al. adds important new data to the debate on hunter-gatherer vs farmer social interaction through exchange, but does not provide new evidence for possible early small-scale animal husbandry.

## Methods

To compare cattle measurements, we used the Logarithmic Size Index (LSI) method. The LSI method measures the logarithmic difference of measurements on fragmented or complete bones compared to the same measurements of a ‘standard’—an individual of known age, sex, and fusion state, thereby allowing observations on intra- and inter-population size variation. By increasing sample size, it addresses the problem of relying too heavily on single specimens judged ‘domesticated’ or ‘wild’. Recent studies continue to show that it is a better method than using direct comparisons of single measurements and works best on breadth measurements^12^. We used all breadth dimensions provided for *Bos* specimens in Ervynck et al. 2016^2^ and Crombé et al. 2020^1^ and breadth dimensions of *Bos* specimens from the Hazendonk culture site of Schipluiden^13^ and available Ertebølle culture and Linearbandkeramik culture sites^13^. We calculated their logarithmic size indices compared to a standard^3^ following the recommendations of Meadow 1999^14^ (Supplementary Data S1).

## Supplementary Information

Supplementary Information.

## Data Availability

The datasets analysed for the current study are available in the EUROEVOL^13^ repository (https://discovery.ucl.ac.uk/id/eprint/1469811/), in Crombé et al. 2020^1^, Ervynck et al. 2016^2^, and Çakırlar et al. 2020^10^ and the data generated are available in the Supplementary Data S1.
